# Does the Health Impact of Exposure to Neighbourhood Green Space Differ between Population Groups? An Explorative Study in Four European Cities

**DOI:** 10.3390/ijerph14060618

**Published:** 2017-06-08

**Authors:** Annemarie Ruijsbroek, Mariël Droomers, Hanneke Kruize, Elise van Kempen, Christopher J. Gidlow, Gemma Hurst, Sandra Andrusaityte, Mark J. Nieuwenhuijsen, Jolanda Maas, Wim Hardyns, Karien Stronks, Peter P. Groenewegen

**Affiliations:** 1 Centre for Nutrition, Prevention and Health Services, National Institute for Public Health and the Environment (RIVM), 3720 BA Bilthoven, The Netherlands; 2 Department of Public Health, Academic Medical Center (AMC), University of Amsterdam, 1100 DD Amsterdam, The Netherlands; m.droomers@amc.uva.nl (M.D.); k.stronks@amc.uva.nl (K.S.); 3 Centre for Sustainability, Environment and Health, National Institute for Public Health and the Environment (RIVM), 3720 BA Bilthoven, The Netherlands; Hanneke.kruize@rivm.nl (H.K.); Elise.van.kempen@rivm.nl (E.v.K.); 4 Centre for Sport, Health and Exercise Research, Staffordshire University, Staffordshire, Stoke-on-Trent ST4 2DF, UK; C.Gidlow@staffs.ac.uk (C.J.G.); G.L.Hurst@staffs.ac.uk (G.H.); 5 Vytauto Didžiojo Universitetas, 44248 Kaunas, Lithuania; S.Andrusaityte@gmf.vdu.lt; 6 ISGlobal, Centre for Research in Environmental Epidemiology (CREAL), E-08003 Barcelona, Spain; mark.nieuwenhuijsen@isglobal.org; 7 University Pompeu Fabra (UPF), 08002 Barcelona, Spain; 8 CIBER Epidemiology y Salud Publica (CIBERESP), 08036 Barcelona, Spain; 9 Department of Social and Organisational Psychology, Faculty of Psychology and Education, Vrije Universiteit (VU), 1081 HV Amsterdam, The Netherlands; jolandamaas@hotmail.com; 10 Department of Criminology, Criminal Law and Social Law, Ghent University, 9000 Ghent, Belgium; Wim.Hardyns@UGent.be; 11 Faculty of Law, University of Antwerp, 2000 Antwerpen, Belgium; 12 NIVEL (Netherlands Institute for Health Services Research), 3500 BN Utrecht, The Netherlands; p.groenewegen@nivel.nl; 13 Department of Human Geography and Department of Sociology, Utrecht University, 3584 CS Utrecht, The Netherlands

**Keywords:** green space, mental health, general health, European cities, subpopulations

## Abstract

It has been suggested that certain residents, such as those with a low socioeconomic status, the elderly, and women, may benefit more from the presence of neighbourhood green space than others. We tested this hypothesis for age, gender, educational level, and employment status in four European cities. Data were collected in Barcelona (Spain; n = 1002), Kaunas (Lithuania; n = 989), Doetinchem (The Netherlands; n = 847), and Stoke-on-Trent (UK; n = 933) as part of the EU-funded PHENOTYPE project. Surveys were used to measure mental and general health, individual characteristics, and perceived neighbourhood green space. Additionally, we used audit data about neighbourhood green space. In Barcelona, there were positive associations between neighbourhood green space and general health among low-educated residents. In the other cities and for the other population groups, there was little evidence that the association between health and neighbourhood green space differed between population groups. Overall, our study does not support the assumption that the elderly, women, and residents who are not employed full-time benefit more from neighbourhood green space than others. Only in the highly urbanised city of Barcelona did the low-educated group benefit from neighbourhood green spaces. Perhaps neighbourhood green spaces are more important for the health of low-educated residents in particularly highly urbanised areas.

## 1. Introduction

Recently, several reviews concluded that contact with green space can benefit health. Strong evidence was found for the short-term restorative effects of contact with nature, suggesting potential health benefits from reductions in chronic stress [1], and for a negative (protective) association between the amount of green space and all-cause mortality [2]. Evidence for the beneficial impact of green on general health, wellbeing, and physical activity was weaker. For mental health, the conclusions differed between reviews, with one concluding that there was strong evidence for a positive relation between the amount of green space and mental health [2] and another concluding there is only weak evidence for this relation [3].

Differences between population groups in their response to green space remain underexplored and have been suggested as an important focus for future research [1,2], especially because the few studies that considered subgroups within the population reported mixed findings [2]. Studying the relation between neighbourhood green space and health for specific population groups is important, because it provides information about who might benefit most from green interventions. Policy makers can use this knowledge to improve the effectiveness of their public health interventions. 

Several studies reported a stronger, positive relationship between green space and health for specific population groups, such as children, older people, housewives, and people with a lower socioeconomic status (SES) [4,5,6,7,8]. It has been suggested that these population groups profit more from a green living environment because they spend more time in their neighbourhood [9] and, therefore, are more exposed to the neighbourhood green space than their counterparts [4,7]. In the case of low SES groups, additional mechanisms have been suggested. Low SES groups might depend more on local green facilities for physical exercise and other activities than higher SES groups, who, because of their better financial situation and higher mobility, are less dependent on the residential proximity to green spaces for the use of these spaces [4,8]. Furthermore, low SES groups might benefit more from local green space because their generally poorer health offers more opportunity for health improvement [10].

As stated, the evidence for the beneficial health impacts of green space for specific population groups is not consistent. The results do not consistently pinpoint the same population groups as those that profit the most and they are inconsistent with regard to different health outcomes. Studies from the United Kingdom, for example, reported that more green space was associated with lower rates of cardiovascular and respiratory diseases in men, but not in women [11]. Moreover, the presence of more green space was associated with better mental health for men at any age, but only for older women [12]. In the Netherlands, associations between neighbourhood green space and health among housewives and the elderly were found for self-reported health symptoms, but not for general and mental health [4]. Another Dutch study reported green space having beneficial (general) health effects in children and the elderly, especially in the most urbanised areas [7]. Furthermore, the health benefits of green space for low SES groups are also not consistently reported, since no SES moderation was found in studies from Spain and the United States [13,14].

The inconsistencies in the literature might reflect that the health benefits of neighbourhood green space not only differ between population groups, but could also depend on specific neighbourhood circumstances, such as the urbanisation level of the area. Therefore, different geographical settings should be investigated using identical measures for green space, health, and demographic characteristics. In the current study, we examined the relationship between green space and health among subpopulations in four European cities: Stoke-on-Trent, United Kingdom; Doetinchem, The Netherlands; Barcelona, Spain; and Kaunas, Lithuania. These four cities differ in size and urbanisation level (see Table 1), but also in climate and societal characteristics (e.g., the employment rate for women, country’s prosperity level). By studying the relationship between neighbourhood green space and health for different subpopulations in these four cities, we wanted to explore the robustness of the associations and whether we can identify circumstances in which green space is associated with the health of different population groups. We thus investigated whether the association between neighbourhood green space and general and mental health differed by the age, gender, educational level, and employment status of residents and if this diversity was consistent across the four European cities. 

## 2. Methods and Materials

Data were collected as part of the PHENOTYPE study, an EU-funded study that examined the health effects of the natural environment and its underlying mechanisms. A survey was carried out from May to October 2013 in four cities. The overall PHENOTYPE project design is reported elsewhere [15].

All subjects gave their informed consent for inclusion before they participated in the study. Ethical approvals were obtained from each of the relevant bodies: Clinical Research Ethics Committee of the Municipal Health Care (CEIC PS-MAR), Barcelona, Spain (2012/4978/I); Staffordshire University Faculty of Health Science ethics committee, United Kingdom; Medical Ethical Committee of the University Medical Centre Utrecht, Netherlands; Lithuanian Bioethics Committee, Lithuania (2012-04-30 Nr.6B-12-147). 

## 3. Study Population and Data Collection

In each city, approximately 30 neighbourhoods varying in the amount of neighbourhood green space and SES were selected (see Table 1 for a description of the neighbourhoods). Neighbourhood green space was defined using Urban Atlas. We extracted the land cover categories from Urban Atlas that seemed most relevant in relation to the aims of our study (i.e., green urban areas; agricultural + semi-natural areas + wetlands; forests; wetlands; water bodies). For Doetinchem, a Dutch database (Top10NL) was used. For neighbourhood SES, country-specific data were used to divide the neighbourhoods into SES categories. See Supplement A for further details on the selection of neighbourhoods based on the Urban Atlas and Top10NL green data and SES data. Survey data were collected using face-to-face interviews, with the exception of Lithuania, where data were collected using a postal questionnaire. Per city, approximately 1000 adults, aged 18–75 years, were interviewed (n = 3947, overall response rate 20%). For further details on the data collection, see Supplementary Materials Supplement A.

Additionally, in each neighbourhood, an audit was carried out to assess the amount and quality of the green spaces and of other characteristics of the neighbourhood. For each neighbourhood, a fixed sample of streets was selected, ensuring that unique, but important, features of the neighbourhood were included. The streets were combined into a route that was systematically inspected by two trained auditors (in a small number of cases, by one auditor). The auditors received a short training and a fieldwork training guide, providing them with specific instructions and example photographs per item. The training also included a shared observation of a neighbourhood where discrepancies between assessments were discussed and resolved before the start of the actual audit. During the audit, the auditors filled out a form containing closed questions about, for instance, the number of trees, the architectural character, maintenance of the area, or the general impression of the neighbourhood. With this approach, we attempted to obtain more objective information about the amount and quality of neighbourhood green. Audit data are considered more objective than the perceptions of residents, but are not as objective as registry or land-use data, since they remain an assessment.

For the purposes of our study, we selected respondents with complete data for the indicators of interest, providing us with a sample of 3771 respondents in 124 neighbourhoods (96% of the study population).

## 4. Measures

### 4.1. Health Measures

#### 4.1.1. Self-Rated Mental Health

Mental health was measured using the Mental Health Inventory (MHI-5), a measure with demonstrable reliability and validity [16]. MHI-5 assesses a person’s feelings of nervousness and depression during the past month, using questions with answers ranging from “all the time” to “never” on a six-point scale. The sum scores of the five answers were transformed into a scale from 0 to 100 [17], with higher scores reflecting better mental health.

#### 4.1.2. Self-Rated General Health

Self-rated general health was measured by a single-question item: “In general, how would you evaluate your health?” The respondents were asked to answer using a five-point Likert-scale (1 = excellent, 2 = very good, 3 = good, 4 = fair, 5 = poor). Responses were dichotomised into excellent/(very) good versus less than good. This measure is a good indicator of the overall health of the population [18]. It has consistently proven to be an independent predictor of mortality [18] and morbidity [19].

### 4.2. Green Space Measures

Measures of assessed and perceived green space were included because previous studies had reported that objective and perceived neighbourhood features might capture different aspects of a neighbourhood, which might differently impact a person’s health [3,20,21]. Furthermore, both amount and quality measures were included, as previous studies had also reported that it was not only the quantity of neighbourhood green space that might be important for health, but also its quality [22,23]. When we refer to the quality of green space, we mean issues like the maintenance of the green feature, its variation, and its attractiveness.

#### 4.2.1. Audit Amount and Quality of Neighbourhood Green Space

Audit data were used to determine the more objective neighbourhood green measurements. The *amount* of neighbourhood green space was based on six items containing information about the percentage of visible gardens, garden size, the arrangement of the gardens, number of trees, size of public green spaces, and size of public blue spaces (Cronbach’s alpha 0.66). The *quality* of neighbourhood green space was determined by the auditor’s answer to the question: “What is your general impression of the quality of the green space in this neighbourhood?” Answers ranged from 1 (very negative) to 5 (very positive). The green indicators were standardised using Z-scores, which were calculated for each city separately. In doing so, neighbourhood green space was compared between the neighbourhoods within one city and not across all cities, making it possible to examine the relative effect of green space on health*.*

#### 4.2.2. Perceived Amount and Quality of Neighbourhood Green Space

The perceived *amount* of neighbourhood green space was measured by asking the respondents: “How would you describe your neighbourhood in terms of green and blue?” Using a five-point Likert scale, the answers ranged from “not at all” (1) to “very” (5). The perceived *quality* of neighbourhood green space was measured by the question: “Overall, in your neighbourhood, how satisfied are you with the quality of the green/blue environment?” Answers were arranged on a five-point Likert scale, with a higher score reflecting a greater satisfaction with the quality. 

We use the term neighbourhood green space for our natural environment measures, because the audit showed that the neighbourhood natural environment consisted foremost of green elements. Moreover, this term corresponds with the terms used in most studies concerning the relation between the natural, green, environment and health.

We conducted ecometric analyses to calculate the average perception of neighbourhood green space [24]. By doing this, we were able to include subjective assessments of neighbourhood green space, while avoiding “same-source bias” (also measured at the same-time) [25,26]. Moreover, with ecometrics, more reliable estimates of the context (i.e., characteristics of places) effect of the neighbourhood can be calculated, because composition (i.e., characteristics of people) effects are taken into account [24]. Ecometric average scores were calculated (stratified by city) and standardised into country-specific Z-scores. See Appendix B for more information about the ecometric approach and for reports on the City-specific reliability scores for the two perceived green measures. 

In Kaunas, the perceived green space indicators had low reliability (Supplementary Materials Supplement B), indicating large differences in the perception of green space between residents of the same neighbourhoods or measurement errors [27]. Because of this, we decided to report the findings for perceived green space in Kaunas in the results section only and to exclude them from the discussion section where the implications of the findings are discussed.

### 4.3. Confounders and Definition of Subpopulations

The individual control variables were sex, age (in years), highest achieved educational level (primary school/no education; secondary school/further education; university degree or higher), nationality (country nationality versus other), employment status (full-time employed versus part-time/unemployed), household composition (children under 12 years: yes/no), and homeownership (yes/no). The neighbourhood SES (low; intermediate; high; based on country-specific data, see Supplementary Materials Supplement A) was included as a neighbourhood level confounder, i.e., a neighbourhood characteristic that may confound the relation between neighbourhood green space and health.

When testing the effect of neighbourhood green space for different age groups, age was divided into two groups (under 65 years versus ≥65 years) to reflect the approximate retirement age in the four countries.

Besides the elderly, women, and low-educated groups (primary school/no education), we tested to see if residents who were not employed full-time profited more from neighbourhood green space than people with a full-time job. We hypothesized that these individuals might be another group of residents who may have been more exposed to neighbourhood green space. The underlying assumption is that residents who are not employed full-time spend more time in the neighbourhood and therefore benefit more from the presence of neighbourhood green space. If we can identify population groups that benefit more from green space in the neighbourhood, this could serve as a basis for policy measures. See Table 2 for the descriptive statistics.

## 5. Analysis

Multilevel regression analyses were applied to take into account the clustering of the data. Level 1 represented the individuals; Level 2: neighbourhoods; Level 3: cities. The city was included as a level in order to assess the city-specific effects in one model. The effects of neighbourhood green space were allowed to vary between different groups of population characteristics (education, age, employment, sex). Analyses were adjusted for demographic characteristics. Furthermore, the analyses using the perceived neighbourhood level of green space measures were adjusted for the individual perception of neighbourhood green space. The neighbourhood green effects were estimated for every city separately, using one model. We tested whether effect modification by age, gender, educational level, and employment status occurred by subtracting the estimates of the subgroups and testing if the differences were statistically significant (*p*-value < 0.05). Significant differences in the estimates between subgroups are reported independent of the significance of the relations between green space and health within each subgroup. Analyses were conducted using SAS 9.3 (SAS Institute Inc., Cary, NC, USA). For a more elaborate description of the analyses, see Supplementary Materials Supplement C. 

## 6. Results

### 6.1. Does the Association between Neighbourhood Green Space and Health Differ by Educational Level?

In Barcelona, with the exception of the audit quality of green space, the association between neighbourhood-level green space and general health differed significantly between low and high-educated groups (Table 3). Low-educated residents reported good general health significantly more often when the amount or quality of neighbourhood-level green was higher. Mental health showed similar patterns, albeit statistically significant differences between the low- and high-educated residents only occurred for the audit amount of neighbourhood green. 

In Doetinchem, there were significant differences between the intermediate- and high-educated residents with regards to the association between the perceived quality of neighbourhood green space and general and mental health. Intermediate-educated residents tended to report better mental and general health when the perceived quality of green space was better, whereas the high-educated residents tended to report poorer health, though neither of these associations were significant. There were no differences between the educational groups in the relation between the amount of green space (neither audit nor perceived) and health. 

In Kaunas, there were significant differences between educational groups in terms of the association between the perceived amount of neighbourhood green and general health. Low-educated residents reported having *poor* general health significantly more often when the perceived amount of neighbourhood green space was higher, while no association was found for the other education groups. In the case of mental health and audit amount of green, there were significant differences between the intermediate- and high-educated residents, in line with Doetinchem and Barcelona. Intermediate-educated residents tended to report better mental health when neighbourhoods were greener, while high-educated residents tended to report poorer mental health (associations not significant).

In Stoke-on-Trent, the relation between the audit quality of green and general health differed significantly between low- and intermediate-educated residents, with the low-educated residents reporting *poor* general health significantly more often when living in neighbourhoods with a better quality of green space. No differences were found between the educational groups for the other green measures or for mental health. 

### 6.2. Does the Association between Neighbourhood Green Space and Health Differ by Age?

In Barcelona and Kaunas, the associations between green space and health differed by age. In Kaunas, the impact of living in greener neighbourhoods (perceived) on general health differed significantly between the age groups in such a way that residents under the age of 65 reported *poor* general health significantly more often in greener neighbourhoods (Table 4). There were also significant differences between the age groups in the relation between green space (audit quality and perceived amount) and mental health, again with residents under the age of 65 tending to report poor health more often, albeit these associations were not significant.

In Barcelona, there was a significant positive association between the perceived amount of neighbourhood green space and general health among the elderly, which was not found in the younger population. No significant differences by age were found for the audit neighbourhood green indicators or for mental health. 

In the other two cities, no differences were found in the relation between neighbourhood-level green space and health between the elderly and the younger age group.

### 6.3. Does the Association between Neighbourhood Green Space and Health Differ by Employment?

Only in Barcelona and only in the case of the audit amount of green space was the hypothesis supported, with a positive association between neighbourhood greenness and mental health among residents who were not employed full-time, and no association among residents with a fulltime job (Table 5). No differences by employment status were found for general health.

In Kaunas, the pattern was reversed, e.g., residents with a full-time job tended to report better mental and general health when the quality of (perceived) green space was better, while those who were not employed in a full-time job tended to report poorer health when the quality of neighbourhood green increased, though neither of these associations were significant. 

In the two other cities, there were no differences in the association between neighbourhood green space and health by employment status.

### 6.4. Does the Association between Neighbourhood Green Space and Health Differ by Gender?

There were no significant differences in the association between neighbourhood green space on the mental or general health of men or women in the four cities (data not shown).

## 7. Discussion

We found some evidence that the low-educated residents benefitted from green space in their neighbourhood as compared to the high-educated residents. The difference in the relationship between green space and health by education was most evident in Barcelona, and to a much lesser extent in Doetinchem. We found no clear evidence for a different relationship between neighbourhood green space and health for people with a different employment status, different age groups, or between men and women. 

### 7.1. Study Limitations

A limitation of this study was the low overall response rate, especially in Doetinchem, the Netherlands. A non-response analysis in the Dutch city showed that the non-respondents reported worse general health than the respondents. This could have led to an underestimation of the health effect of green space for the low educated group in Doetinchem. Another limitation in all four cities, but particularly in Doetinchem and Kaunas, is the underrepresentation of low-educated people. In these two cities, the almost absent residents with a low-educational level (1.2% and 1.7%, respectively, Table 2) could have complicated the demonstration of significant associations between neighbourhood green and health for this population group. Furthermore, our audit measure of the quality of neighbourhood green space has limitations. It was based on a single- and broadly described item (”general impression”). This might not have assessed the quality of neighbourhood green space accurately enough, which may have hindered the assessment of the relation with health. Furthermore, we cannot completely rule out that, despite the use of identical measurements, data might not be comparable because of cultural differences in the interpretation of survey and audit questions. This should be kept in mind when interpreting the results. Finally, a limitation of the study was that cities and countries overlapped. This made it impossible to determine whether the differences between the four settings were indeed city differences or could have been country (national) differences. Additional analyses showed that there were differences between the cities in the appreciation of nearby green and blue space. The residents in Barcelona and in Stoke-on-Trent more often reported nearby green/blue to be very important for physical activity, relaxation, and social activities than the residents of Doetinchem (in the case of social activity and relaxation) and Kaunas (in all cases). On the other hand, nearby green for walking and biking paths for commuting was found to be most important among the residents in Doetinchem, which might be related to the fact that active transport, in particular cycling, is more common in The Netherlands than in the other countries. These findings indicate that there may indeed be cultural differences between the cities or countries in the appreciation of the natural environment near the home, and that these differences might have influenced our findings. Therefore, future international comparison studies should preferably avoid the overlap between cities and countries. 

### 7.2. Interpretation and Implications of the Study’s Findings

This study found no evidence that employment status did interfere with the association between neighbourhood green space and health. The underlying assumption that residents who are not employed full-time spend more time in the neighbourhood and therefore benefit more from the presence of neighbourhood green space may be incorrect. It is possible that people who are not employed full-time and have more leisure time do not spend that extra time in their own neighbourhood. Future studies that investigate the hypothesis of differences in exposure to neighbourhood green space should specifically measure the amount of time that respondents spend in various locations, for example, by using Space-Time Budget methods [28,29]. Space-Time Budget methods provide information within a specific period where people are (at what geographical location), under what circumstances (for instance with whom), and what they are doing [29]. This type of information can provide more insight into the actual exposure to green space, both inside and outside the neighbourhood, and its effect on the health of different population groups. The absence of a differential relation between green space and health by employment status could, however, also be the result of the broad classification of employment status used in our study where the group “not full-time employed” consisted of individuals with a part-time job, those who were unemployed, retired, and who carried out volunteer work.

We found no evidence that the association between neighbourhood green space and health was different for men and women, although this has been reported in previous studies [4,5,11,12], and for other neighbourhood characteristics [30]. We, and others, hypothesized that women are more exposed to neighbourhood green space since they are often the primary caregivers who supervise the children and work part-time more often than men do. They are therefore likely to spend more time in the direct living environment. It has been suggested that because the social roles that are typically fulfilled by women are more locally orientated, women’s health is more strongly related to the characteristics of the neighbourhood (including green space availability) than men’s health [30]. In Doetinchem and Stoke-on-Trent, women worked full-time less often than men did, but we found no differential effect for gender in these cities. It has also been argued that the health benefits of green space for women are more closely associated with subjective green quality indicators than is the case for men (Richardson and Mitchell, 2010). Our results regarding both the quantity and quality measures of green space do not support this hypothesis. Nor did we find evidence that the perception of neighbourhood green space is more important for women’s health and objective measures of green space than for the health of men.

In the four cities, by testing multiple green indicators, we found only two instances where the relation between neighbourhood green space and health differed significantly between the age groups. In Barcelona, the general health of the elderly benefitted from the perception of neighbourhood greenness, whereas the general health of the residents under the age of 65 did not, in concordance with earlier studies from the Netherlands [4,7]. In Kaunas, the association between the audit quality of green space and mental health differed between the age groups, with residents under the age of 65 tending to report poor mental health more often. Overall, we found no support that neighbourhood green space specifically benefits the health of the elderly.

Lower educated residents seem to benefit more from neighbourhood green space than high-educated residents in Barcelona, and to a far lesser degree, in Doetinchem. This has been reported in previous studies [4,6,7], though not consistently [13,14]. It is not clear which underlying mechanism plays a role. The hypothesis that certain population groups such as the low-educated are more exposed to the neighbourhood environment and therefore benefit more from neighbourhood green space needs more precise examination, since most studies, ours included, do not directly study the differences in exposure to green space between population groups. If the health benefits resulted from the mechanism that low-educated groups are generally in worse health, which offers them more room for health improvement, this leaves the question of why this mechanism does not apply to the low-educated residents in Kaunas and Stoke-on-Trent, where the association between the low-educated group and neighbourhood green space was not found. Perhaps the geographical setting played a role. In our study, the different patterns regarding neighbourhood green space and health between subpopulations were not consistent within or across the four urban settings. Most relations between green space and health were found in Barcelona, which is by far the largest and most densely populated city in our study (see Table 1). We therefore hypothesize that particularly in highly urban circumstances, neighbourhood green space is important for the health of particularly low-educated residents. This suggests that the health impact of neighbourhood green space is not only confined to certain subpopulations, but also depends on the residential circumstances, in this case, urbanicity. However, this hypothesis needs further examination. Overall, our findings show that the patterns regarding neighbourhood green space and health between subpopulations differed between the four cities and that is important to consider the local context before research findings are translated into policy recommendations or interventions.

## 8. Conclusions

Overall, our study does not support the assumption that the elderly, women, and residents who are not employed full-time benefit more from neighbourhood green space than others. The finding that the health benefits of green spaces for lower educated groups were only found in Barcelona might indicate that only in highly urbanised areas neighbourhood green spaces are important for the health of low-educated residents and not for higher educated neighbours.

## Figures and Tables

**Table 1:** Geographical information about the four PHENOTYPE cities and their neighbourhoods.

Information Concerning the Spatial Units Used for Neighbourhood Selection in Each City				
	Doetinchem	Barcelona	Stoke-on-Trent	Kaunas
Spatial unit	Neighbourhoods	Census Areas	Lower Super Output Areas	Voting Districts
Count of spatial units	83	1061	241	116
Average population size of a spatial unit	670	1538	1508	3400
Average surface area (SD) in km^2^ of a spatial unit	0.96 (1.22)	0.11 (0.64)	1.26 (4.22)	1.34 (1.85)
Average population density (pers./km^2^)	697	13,981	1196	2537

Doetinchem is a medium-sized city, situated in the eastern part of the Netherlands. The city included 56,247 inhabitants and covers a surface area of 80 km^2^ (in 2012). Barcelona is the second-largest city in Spain, has 1,631,259 inhabitants, and covers a surface area of 102 km^2^ (in 2011). Stoke-on-Trent is a city in the heart of England and is made up of multiple towns with a total surface area of 304 km^2^ and 363,421 inhabitants (in 2010). Kaunas, with 319,213 inhabitants and a surface area of 156 km^2^, is the second-largest city in Lithuania (in 2011).

**Table 2:** Descriptive statistics of the respondents by city.

	Doetinchem–The Netherlands			Barcelona–Spain			Stoke-on-Trent–UK			Kaunas–Lithuania		
Total n	847			1002			933			989		
	%	Mean (SD)	Range	%	Mean (SD)	Range	%	Mean (SD)	Range	%	Mean (SD)	Range
Mental health (continuous, 1–100)		80.2 (13.5)	20–100		71.0 (15.9)	12–100		73.8 (16.3)	8–100		70.8 (16.9)	8–100
(very) good general health	96.2			85.2			77.5			38.5		
Sex–male	43.2			47.0			48.0			39.5		
*Educational level (three categories)*												
Primary school,	1.2			14.5			9.4			1.7		
Secondary school/further education	47.2			38.3			64.0			26.3		
Higher education/university or up	51.6			47.2			26.6			72.0		
Employment status—employed full-time	27.7			43.0			43.0			34.6		
Age (continuous)		56.4 (12.2)	19–75		45.0 (15.6)	18–75		46.0 (16.1)	18–75		59.7 (13.8)	18–75
*Age (two categories)*												
Under 65	70.8			85.2			82.9			55.5		
65 and older	29.2			14.8			17.1			44.5		
Homeowner	22.6			57.3			59.1			89.2		
Nationality–country nationality	96.0			76.0			95.0			96.2		
Child(ren) under twelve in the household	15.1			18.6			21.7			4.9		

**Table 3:** Multilevel regression analysis for the relationship between neighbourhood-level green space (standardised estimates) and mental health (MH) and general health (GH) (standard errors in parentheses), by education (the *p*-value is provided for significant differences between subgroups) ^a^.

	Doetinchem–The Netherlands		Barcelona–Spain		Stoke-on-Trent–UK		Kaunas–Lithuania	
	MH	GH	MH	GH	MH	GH	MH	GH
Audit amount green								
Low-educated	−11.782 (7.08)	1.442 (1.27)	3.859 (1.53) *	0.794 (0.28) **	−1.455 (2.09)	0.350 (0.30)	2.206 (3.45)	−0.811 (0.55)
Intermediate-educated	0.874 (0.98)	0.211 (0.28)	1.835 (0.98)	0.159 (0.18)	1.271 (0.83)	−0.130 (0.13)	1.645 (1.12)	0.019 (0.16)
High-educated	0.200 (0.90)	−0.409 (0.29)	0.604 (0.85)	0.305 (0.19)	−0.075 (1.04)	−0.063 (0.19)	−0.804 (0.81)	−0.079 (0.11)
*p-value difference*			*0.049*	*0.048*			*0.038*	
Audit quality green								
Low-educated	−0.630 (4.69)	−0.542 (2.33)	0.604 (1.69)	0.540 (0.26) *	−1.103 (2.07)	−0.640 (0.31) *	0.317 (3.92)	0.275 (0.65)
Intermediate-educated	1.036 (1.00)	0.548 (0.24) *	0.194( 0.99)	0.061 (0.17)	−0.260 (0.84)	0.090 (0.12)	−0.728 (1.09)	−0.064 (0.15)
High-educated	−0.197 (0.99)	−0.273 (0.36)	0.174 (0.86)	0.354 (0.19)	0.479 (1.11)	0.060 (0.20)	−0.659 (0.82)	−0.086 (0.11)
*p-value difference*						*0.024*		
Perceived amount green ^b^								
Low-educated	0.365 (4.06)	−0.365 (1.39)	2.850 (1.55)	0.820 (0.25) ***	−2.486 (1.81) *	−0.238 (0.27)	0.905 (4.25)	−1.886 (0.90) *
Intermediate-educated	1.534 (0.94)	0.250 (0.26)	0.509 (0.97)	0.212 (0.17)	1.429 (0.85)	−0.000 (0.13)	−0.001 (1.13)	−0.007 (0.16)
High-educated	0.473 (0.83)	−0.315 (0.31)	0.715 (0.83)	0.215 (0.17)	1.459 (1.20)	0.129 (0.23)	0.550 (0.77)	−0.119 (0.10)
*p-value difference low–intermediate*				*0.034*				*0.038*
*p-value difference low–high*				*0.038*				*0.049*
Perceived quality green ^b^								
Low-educated	−0.466 (4.87)	−1.826 (1.96)	2.528 (1.38)	0.686 (0.23) **	−0.127 (1.70)	−0.060 (0.24)	3.986 (4.14)	−0.978 (0.65)
Intermediate-educated	1.621 (0.92)	0.379 (0.25)	0.258 (0.95)	0.130 (0.16)	0.877 (0.86)	−0.061 (0.13)	−1.606 (1.12)	0.003 (0.16)
High-educated	−0.639 (0.84)	−0.477 (0.32)	0.499 (0.91)	0.285 (0.18)	0.333 (1.17)	0.159 (0.22)	0.170 (0.79)	−0.065 (0.10)
*p-value difference*	*0.027*	*0.036*		*0.048*				

^a^ adjusted for age, sex, ethnicity, household composition, employment status, homeownership, neighbourhood SES. ^b^ adjusted for individual deviation from the neighbourhood level perceived green score. * significant associations between green and health (* *p*-value < 0.05, ** *p*-value < 0.01, *** *p*-value < 0.001). *p*-value difference: significant differences between the effect estimates of the subgroups.

**Table 4:** Multilevel regression analysis for the relationship between neighbourhood green space (standardised estimates) and mental health (MH) and general health (GH) (standard errors in parentheses), by age (for significant differences between subgroups the *p*-value is provided) ^a^.

	Doetinchem–The Netherlands		Barcelona–Spain		Stoke-on-Trent–UK		Kaunas–Lithuania	
	MH	GH	MH	GH	MH	GH	MH	GH
Audit amount green								
18–65 years	0.788 (0.84)	−0.246 (0.27)	1.389 (0.73)	0.319 (0.14) *	0.787 (0.75)	−0.065 (0.12)	−0.671 (0.95)	−0.227 (0.13)
65 and older	−0.439 (1.15)	0.157 (0.31)	1.792 (1.39)	0.529 (0.25) *	0.033 (1.50)	−0.005 (0.21)	0.403 (0.87)	0.035 (0.12)
*p-value difference*								
Audit quality green								
18–65 years	0.734 (0.88)	0.227 (0.27)	−0.116 (0.75)	0.328 (0.14) *	−0.415 (0.77)	−0.004 (0.12)	−1.664 (0.88)	−0.108 (0.12)
65 and older	−0.429 (1.16)	0.190 (0.29)	2.224 (1.37)	0.146 (0.23)	2.354 (1.54)	0.177 (0.22)	0.444 (0.93)	−0.097 (0.13)
*p-value difference*							*0.041*	
Perceived amount green ^b^								
18–65 years	0.861 (0.78)	0.095 (0.26)	0.528 (0.73)	0.228 (0.13)	0.712 (0.79)	−0.027 (0.12)	−0.626 (0.85)	−0.236 (0.12) *
65 and older	0.855 (1.15)	−0.144 (0.33)	3.010 (1.37) *	0.756 (0.23) **	2.804 (1.42) *	0.248 (0.20)	1.719 (0.91)	0.083 (0.13)
*p-value difference*				*0.033*			*0.018*	*0.026*
Perceived quality green ^b^								
18–65 years	0.491 (0.77)	0.090 (0.25)	0.386 (0.74)	0.227 (0.13)	0.134 (0.79)	−0.023 (0.12)	−0.367 (0.86)	−0.153 (0.12)
65 and older	−0.219 (1.14)	−0.113 (0.31)	2.890 (1.41) *	0.687 (0.23) **	2.403 (1.36)	0.212 (0.19)	−0.088 (0.91)	0.008 (0.13)
*p-value difference*								

^a^ adjusted for sex, education, ethnicity, household composition, employment status, homeownership, neighbourhood SES. ^b^ adjusted for individual deviation from the neighbourhood level perceived green score.* significant associations between green and health (* *p*-value < 0.05, ** *p*-value < 0.01). *p*-value difference: significant differences between the effect estimates of the subgroups.

**Table 5:** Multilevel regression analysis for the relationship between neighbourhood green space (standardised estimates) and mental health (MH) and general health (GH) (standard errors in parentheses), by employment (for significant differences between subgroups the *p*-value is provided) ^a^.

	Doetinchem–The Netherlands		Barcelona–Spain		Stoke-on-Trent–UK		Kaunas–Lithuania	
	MH	GH	MH	GH	MH	GH	MH	GH
Audit amount green								
Full-time	−0.064 (0.1.20)	−0.089 (0.53)	0.161 (0.88)	0.555 (0.21) **	0.921 (0.90)	−0.037 (0.17)	0.051 (1.09)	−0.077 (0.14)
Not full-time	0.617 (0.82)	−0.050 (0.22)	2.546 (0.84) **	0.254 (0.14)	0.391 (0.89)	−0.079 (0.13)	−0.105 (0.81)	−0.064 (0.11)
*p-value difference*			*0.016*					
Audit quality green								
Full-time	−0.571 (1.17)	0.222 (0.50)	−0.659 (0.91)	0.342 (0.21)	−1.187 (0.94)	−0.063 (0.17)	−1.756 (1.03)	0.154 (0.14)
Not full-time	0.813 (0.89)	0.276 (0.22)	0.986 (0.86)	0.228 (0.14)	0.785 (0.87)	0.055 (0.13)	−0.135 (0.82)	−0.214 (0.11)
*p-value difference*								
Perceived amount green ^b^								
Full-time	0.661 (1.03)	−0.144 (0.52)	0.121 (0.88)	0.333 (0.19)	0.286 (0.97)	−0.105 (0.18)	0.803 (0.96)	−0.152 (0.12)
Not full-time	1.035 (0.81)	0.041 (0.22)	1.508 (0.83)	0.332 (0.14) *	1.613 (0.90)	0.039 (0.13)	0.208 (0.82)	−0.080 (0.11)
*p-value difference*								
Perceived quality green ^b^								
Full-time	−0.316 (1.03)	−0.009 (0.47)	−0.076 (0.93)	0.514 (0.17) **	−0.360 (0.97)	−0.025 (0.17)	1.174 (0.98)	0.120 (0.13)
Not full-time	0.646 (0.80)	0.020 (0.21)	1.301 (0.83)	0.228 (0.13)	1.204 (0.89)	−0.013 (0.12)	−0.949 (0.83)	−0.163 (0.10)
*p-value difference*							*0.032*	*0.045*

^a^ adjusted for age, sex, education, ethnicity, household composition, homeownership, neighbourhood SES. ^b^ adjusted for individual deviation from the neighbourhood level perceived green score. * significant associations between green and health (* *p*-value < 0.05, ** *p*-value < 0.01). *p*-value difference: significant differences between the effect estimates of the subgroups.

## Supplementary Materials

The following are available online at www.mdpi.com/1660-4601/14/6/618/s1, Supplement A: Data collection strategy and response rate per city, Supplement B: Ecometrics method to aggregate individual perception to the neighbourhood level, Supplement C: Technical description of the analyses.
