# Supplementary material for: Does the Health Impact of Exposure to Neighbourhood Green Space Differ between Population Groups? An Explorative Study in Four European Cities
Source: Int J Environ Res Public Health. 2017 Jun 8;14(6):618. doi: 10.3390/ijerph14060618 (PMC5486304; doi:10.3390/ijerph14060618)
Supplement: Supplementary file 1 [file ijerph-14-00618-s001.docx]

**Supplementary materials**

Does the Health Impact of Exposure to Neighbourhood Green Space Differ between Population Groups? An Explorative Study in Four European Cities

Annemarie Ruijsbroek, Mariel Droomers, Hanneke Kruize, Elise van Kempen, Christopher J. Gidlow, Gemma Hurst, Sandra Andrusaityte, Mark J. Nieuwenhuijsen, Jolanda Maas, Wim Hardyns, Karien Stronks and Peter P. Groenewegen

**Supplement A. Data Collection Strategy and Response Rate per City**

The PHENOTYPE data were derived from face-to-face interviews in The Netherlands, United Kingdom and Spain. In Lithuania, data were collected with a postal questionnaire. A city-specific 5*3 cross table was produced, fitting in all neighbourhoods according to a categorization by green (5 categories) and SES (3 categories). Neighbourhood SES was country-specific. For Doetinchem, the average monthly household income was used. For Stoke-on-Trent, the English indices of deprivation 2010 (IMD 2010) was used, which included data from 7 domains (income deprivation, employment deprivation, health deprivation and disability, education skills and training deprivation, barriers to housing and services, living environment deprivation, and crime). For Barcelona, a deprivation index from the MEDEA project was used, which included information about education and (un)employment. For Kaunas, a combination of education level and income from a Kaunas Citizen sample was used. Based on the tertiles of the country specific distribution of SES, three categories of SES were defined (low, intermediate and high SES level). Neighbourhood green was defined using Urban Atlas. For Doetinchem, Urban Atlas was not available and data of a Dutch database (Top10NL) were used. The straight-line distance to green spaces larger than 1 hectare was calculated for all residential addresses (households) within each neighbourhood, using GIS. Subsequently, the averaged distances were ranged into quintiles to define the five green categories. From each table cell from the cross table, two neighbourhoods with sufficient adult population were selected.

In Doetinchem, a total of 10,220 residents were approached by mail after a random selection of addresses. In total, 861 people participated (8.4% response rate). In Stoke-on-Trent, 2,826 randomly selected addresses were sent a letter. Interviewers then visited addresses in each neighbourhood in a random order. At each address, interviewers used the birthday rule (next birthday) to randomly select the individual per household. In total, 1,044 people participated (36.9% response rate). In Barcelona, 11,543 people were selected at random across the 30 neighbourhoods. The selected people were distributed in candidates and substitutes (at the rate of 1/10), ensuring that each candidate had 10 substitutes with matching age range, sex and neighbourhood. A letter of invitation was sent. Interviewers then visited the addresses of the candidates. If the candidate was not contactable or not willing to participate, then the interviewers approached the first substitute (and so on). In total, 2,230 people were contacted of which 1,045 participated (46.9% response rate). Finally, in Kaunas, 5,840 adults aged 20-75 were randomly selected from a 2006-2009 survey. Because of change in addresses 1,168 people could not be reached. Consequently, 4,672 people were invited by mail to fill out a postal questionnaire. This postal questionnaire was sent in the same period the face-to-face interviews took place in the other cities. In total, 997 people participated (21.3% response rate).

**Supplement B. Ecometrics Method to Aggregate Individual Perception to the Neighbourhood Level**

Ecometics was used to calculate our neighbourhood measures from the survey data. With ecometrics, more reliable estimates of the context effect of the neighbourhood can be calculated by accounting for composition effects. We adjusted the aggregated measures for six individual characteristics that may influence the perception of the neighbourhood characteristics in question; sex, age, educational level, ethnicity, employment status, household composition and homeownership. Additionally, we adjusted for owning a dog in the multilevel models for neighbourhood greenness, to account for the assumption that dog owners have better knowledge of the amount and quality of the green spaces in their neighbourhood.

To aggregate the green indicators a two-level (respondents and neighbourhoods) linear regression model was used, because we only included one green item at the item level. The residuals of the neighbourhood measurement, i.e. the part that cannot be attributed to participants’ response patterns and measurement error, constitutes the neighbourhood greenness measurement. Positive values indicate higher than average levels of neighbourhood greenness. Reliability scores can be calculated using the formula by Hox (2010):

*ë_j_* = *σ*^2^*_neighbourhood_* / [*σ*^2^_neighbourhood_ + [*σ*^2^_individual_ / *n_j_*]]

*ë_j_* is the reliability of the neighbourhood measure*. σ*^2^*_neighbourhood_* is the variance between neighbourhoods; *σ*^2^_individual_ is the variance between individuals within the neighbourhoods; *n_j_* is the mean number of respondents per neighbourhood. The reliability *ë_j_* is close to 1 when group sizes are large and/or the variability of the intercepts across the groups in comparison to the individual variability is large. The reliability *ë_j_* is close to 0 when group sizes are small or when there is little variation across groups compared to the individual variation (Hox, 2010).

The reliability scores for the green measures for each city

|  | **Doetinchem** | **Barcelona** | **Stoke-on-Trent** | **Kaunas** |
| --- | --- | --- | --- | --- |
| Reliability score perceived amount green | *0.78* | *0.95* | 0.81 | 0.10 |
| Reliability score perceived quality green | *0.58* | *0.91* | 0.78 | 0.30 |

**Supplement C. Technical Description of the Analyses**

We used multilevel regression models for our analyses. City was included as separate level, in order to adjust for intercept differences between the four cities. This resulted in a three level regression model: level 1 representing the individuals, level 2 the neighbourhoods, and level three the cities.

All models are corrected for individual level (age, gender, education, ethnicity, household composition, employement status, homeownership, individual perceived green measurement) confounders and one neighbourhood (neighbourhood SES) confounder. In case of the perceived green measurements, the individual perceived green measurements (scored as deviations from the neighbourhood mean perceived green measurements) are added as confounders to control for the potential relation between the outcome and the individual perceived green measurement, this makes the relation between the outcome and the average neighbourhood perceived green measurements more comparable to the objective neighbourhood green measurement. The effects of interest are the different neighbourhood green measurements.

All the neighbourhood green effects are estimated simultaneously for the four cities separately. This allows for the comparison (and testing the difference in regression coefficients) of the, same, green effects between cities. Next the green effects per city are allowed to vary for different groups of people (age, employement, education). This allows the comparison of the green effects for different groups within and between cities.

Model with the green effects allowed to vary between cities.

$Y_{ijk}=\beta_{0}+\sum_{h=1}^{h=n} \beta_{h}X_{ijk}+\beta Z_{jk}+\sum_{g=1}^{g=m} \sum_{c=1}^{c=4} \beta_{gc}Z_{jk}+\omega_{k}+\mu_{jk}+\epsilon_{ijk}$

-$Y_{ijk}$: health outcome, for individual I in neigbourhood j in city k

-$\beta_{0}$: intercept

-$\sum_{h=1}^{h=n} \beta_{h}X_{ijk}$: confounders (1 to n) measured on the level of the individual

-$\beta Z_{jk}$: one confounder measured at neighbourhood level

-$\sum_{g=1}^{g=m} \sum_{c=1}^{c=4} \beta_{gc}Z_{jk}$: Z = green variables (g, 1 to m) for every city (c, 1 to 4)

g= neighbourhood green measurement

c=indicator variable for a city (0=not belonging to that city,

1=belonging to that city)

Z=g*c

Note that for every city this variable is added to the model

-$\omega_{k}$ : between city variance

-$\mu_{jk}$ : between neighbourhood variance

-$\epsilon_{ijk}$ : individual level error variance

Model with the green effects allowed to vary between cities for different population groups.

$Y_{ijk}=\beta_{0}+\sum_{h=1}^{h=n} \beta_{h}X_{ijk}+\beta Z_{jk}+\sum_{g=1}^{g=m} \sum_{c=1}^{c=4} \sum_{p=1}^{p=q} \beta_{gcp}Z_{jk}+\omega_{k}+\mu_{jk}+\epsilon_{ijk}$

-$Y_{ijk}$: health outcome, for individual I in neigbourhood j in city k

-$\beta_{0}$: intercept

-$\sum_{h=1}^{h=n} \beta_{h}X_{ijk}$: confounders (1 to n) measured on the level of the individual i

-$\beta Z_{jk}$: one confounder measured at neighbourhood level j

-$\sum_{g=1}^{g=m} \sum_{c=1}^{c=4} \sum_{p=1}^{p=q} \beta_{gcp}Z_{jk}$:Z = green variables (g, 1 to m) for every city (c, 1 to 4) for different

population groups (p, 1 to q groups)

g = neighbourhood green measurement

c = indicator variable for a city (0=not belonging to that city,

1 = belonging to that city)

P = indicator variable for population group (0= not belonging to that group, 1=belonging to that group)

Z=g*c*p

Note that for every city and every population group this variable is added to the model

-$\omega_{k}$ : between city variance

-$\mu_{jk}$ : between neighbourhood variance

-$\epsilon_{ijk}$ : individual level error variance

The intraclass correlations (ICC) at the neighbourhood level were calculated for general and mental health based on the empty models. The ICC estimates are the proportion of variation in general and mental health between residents that is related to neighbourhood characteristics.

The intraclass correlation (ICC) for each city (%).

|  | **Doetinchem** | **Barcelona** | **Stoke-on-Trent** | **Kaunas** |
| --- | --- | --- | --- | --- |
| ICC mental health | 0.51 | 6.71 | 8.51 | 0.69 |
| ICC general health | 3.98 | 4.72 | 6.86 | 5.14 |
